# Engineered myocardial tissues constructed *in vivo *using cardiomyocyte-like cells derived from bone marrow mesenchymal stem cells in rats

**DOI:** 10.1186/1423-0127-19-6

**Published:** 2012-01-12

**Authors:** Yujie Xing, Anlin Lv, Li Wang, Xuebo Yan, Wei Zhao, Feng Cao

**Affiliations:** 1 Department of Cardiology, Xijing Hospital, Fourth Military Medical University, Xi'an, Shaanxi, 710032, China

**Keywords:** Polylactic acid-co-glycolic acid, Bone marrow mesenchymal stem cells, Engineered myocardial tissue

## Abstract

**Background:**

To explore the feasibility of constructing engineered myocardial tissues (EMTs) *in vivo*, using polylactic acid -co-glycolic acid (PLGA) for scaffold and cardiomyocyte-like cells derived from bone marrow mesenchymal stem cells (BMMSCs) for seeded cells.

**Methods:**

BMMSCs were isolated from femur and tibia of Sprague-Dawley (SD) rats by density-gradient centrifugation. The third passage cells were treated with 10 μmol/L 5-azacytidine (5-aza) and 0.1 μmol/L angiotensin II (Ang II) for 24 h, followed by culturing in complete medium for 3 weeks to differentiated into cardiomyocyte-like cells. The cardiomyocyte-like cells were seeded into PLGA scaffolds to form the grafts. The grafts were cultured in the incubator for three days and then implanted into the peritoneal cavity of SD rats. Four weeks later, routine hematoxylin-eosin (HE) staining, immunohistochemical staining for myocardium-specific cardiac troponin I (cTnI), scanning electron microscopy and transmission electron microscopy were used to analyze the morphology and microconstruction of the EMTs in host rats.

**Results:**

HE staining showed that the cardiomyocyte-like cells distributed equally in the PLGA scaffold, and the nuclei arranged in the spindle shape. Immunohistochemical staining revealed that majority of engrafted cells in the PLGA -Cardiomyocyte-like cells group were positive for cTnI. Scanning electron microscopy showed that the inoculated cells well attached to PLGA and grew in 3 dimensions in construct. Transmission electron microscopy showed that the EMTs contained well arranged myofilaments paralleled to the longitudinal cell axis, the cells were rich in endoplasmic reticulum and mitochondria, while desmosomes, gap junction and Z line-like substances were also can be observed as well within the engrafted cells.

**Conclusion:**

We have developed an in vivo method to construct engineered myocardial tissue. The *in vivo *microenvironment helped engrafted cells/tissue survive and share similarities with the native heart tissue.

## Background

Ischemic heart disease and dilated cardiomyopathy are the major causes of morbidity and mortality worldwide. The limited capability of the surviving cardiomyocytes to proliferate render the damaged heart susceptible to unfavorable remodeling processes and morbid sequelae such as heart failure [1]. Established pharmacologic treatment can decelerate but not stop the progression of heart failure once a significant part of the myocardium tissue is damaged. Currently heart transplantation is the only viable treatment option for end-stage heart failure patients. Given the persistent shortage of donor heart organs' donation, stem cell therapy has emerged as a promising cell resources for healing final stage heart disease because it provides a virtually ideal source of cardiomyocytes, endothelial cells, and other differentiated cell types.

The efficacy of stem cell transplantation has been demonstrated by many animal and clinical studies. Different cell types have been proposed for cardiac regeneration, including embryonic stem cells, skeletal myoblasts, endothelial progenitor cells and bone marrow mononuclear cells [2-4]. In these cell types, bone marrow mesenchymal stem cells (BMMSCs) have gained attention as an easily accessible, homogeneous cell population for cardiac repair. Tissue engineering with stem cell, aiming at restoring, maintaining or enhancing tissue and organ function, provides a potential therapeutic alternative to whole organ transplantation and offers the possibility of creating functional tissue equivalents for scientific studies and tissue repair [5].

Myocardial tissue engineering is an emerging technique that combines cells with scaffolding polymers in an attempt to generate functional 3-dimensional tissues outside of the body that can be tailored in size, shape, and function according to the respective needs before implantation and improve heart function by integrating cardiomyocytes into the native organ architecture [6-11]. Generally speaking, there are two main methods for myocardial tissue engineering: one is directly injecting cardiomyocytes or cardiac-like cells into heart. More recently, grafting techniques for single cells have been established. Many researchers have demonstrated that BMMSCs implantation induces myocardial regeneration and improves cardiac function through myogenesis and angiogenesis [12-14]. However, cell implantation studies are generally hampered by low cell retention and survival as well as a restricted cardiomyogenic potential in most of the applied cells [15-18]. Another method is the transplantation of three-dimensional cardiac grafts. Li et al used a preformed biodegradable gelatin mesh to reconstitute cardiac myocytes in a three-dimensional structure [19]. Recent advances in cell sheet tissue engineering have enabled treatment of cardiac failure through the transplantation of cell sheets in animal models [20]. In present study, we have used a different approach to construct engineered myocardial tissue using cardiomyocyte-like cells derived from bone marrow mesenchymal stem cells and PLGA scaffold. The technique employed PLGA to support the endogenous capability of induced cells to form a heart tissue-like structure.

## Methods

polyclonal mouse anti-a-actin (sarcomeric, Boster), polyclonal goat anti-troponin I (cTnI, Santa Cruz), polyclonal rabbit anti-connexin 43 (anti-Cx43, Boster), anti-goat FITC-conjugated IgG (Boster), anti-rabbit FITC-conjugated IgG (Boster), anti-mouse Cy3-conjugated IgG (Boster), Hoechst33258 (Sigma, USA), dimethyl sulfoxide (DMSO, Sigma, USA)

### Methods

#### Isolation and culture of BMMSCs

Bone marrow was aspirated from femur and tibial bones of 4-week-old male Sprague-Dawley (SD) rats (approximately 100 g) following dislocation. The marrow were collected and diluted with 4 mL of Dulbecco's modified Eagle's medium (DMEM, Hyclone, USA), mixed with an equal volume of 1.073 g/ml Percoll solution (Sigma, USA), and then centrifuged at 1500 g for 15 min. The enriched cells were collected from the interphase and then re-suspended in culture medium. The cells were plated in a 25 cm^2 ^flasks and grown in complete DMEM-low glucose medium supplemented with 10% fetal bovine serum (Gibco, USA), 100 U/mL penicillin, 100 μg/mL streptomycin,300 mg/l L-glutamine and 10 mmol/L HEPES at 37°C in a humidified atmosphere of 5% CO2. After 3 days, non-adherent hematopoietic cells were discarded and the adherent cells were washed twice with PBS. The culture medium was replenished every 3 days. When the density of cell colonies had reached approximately 90% confluence, the cells were trypsinized (0.25% trypsin) and transferred to fresh flasks at 1:2 ratio.

#### Differentiation of cardiomyocyte-like cells

BMMSCs at passage 3 were induced to differentiation via incubating with complete medium containing 10 mM 5-azacytidine (Sigma, USA) and 0.1 mM angiotensin II (Sigma, USA) for 24 h. Afterwards, the cells were washed 3 times with phosphate buffer solution and the medium was changed to complete medium without 5-aza and AngII [21]. The medium was changed every 3 days. The experiment terminated 3 weeks after the drug treatment, then the cells were prepared for the following experiments.

#### Scaffolds preparation

The scaffolds consisted of 50%-polylactic acid (PLA) and 50%-polyglycolic acid (PGA). The molecular mass of PLA and PGA were 20 kDa. The porous microstructure was created as described by using a particulate leaching method with salt grains of 150-200 μm in diameter; resulting in 90% porosity. The sponges were sliced into squares (10 mm×10 mm×1 mm). Before cell seeding, the scaffolds were sterilized by Co^60 ^irradiation, immersed in phosphate buffer saline for 1 h, and then in DMEM for 1 h. The cardiomyocyte-like cells were trypsinized for 2 to 3 minutes.2 mL of cell culture medium was added to stop the digestion. After being pipetted, the suspension was collected in a 15-mL tube. The cell number was determined with a cell counter. The tube containing the cell suspension was centrifuged for 5 minutes at 1000*g*. After the supernatant was removed, the cell pellet was re-suspended in 1 ml of culture medium (5~8 × 10^8 ^cells/ml). The suspension was slowly placed onto the surface of squares of presoaked PLGA scaffolds which were 10 mm in length, 10 mm in width and 1 mm in height. After another 4 hour, 1 mL of culture medium was carefully added to submerge the graft totally. Grafts were incubated at 37°C, in 5% CO_2 _and 95% air for three days. The BMMSCs- scaffold graft were created by the same way. For use as controls, squares of PLGA scaffolds were placed in culture medium without any cell inoculation.

#### Animal Study

Thirty-six Male Sprague-Dawley rats (250-300 g) were randomly assigned into three groups: the PLGA -Cardiomyocyte-like cells group (PC group) received implantation of cardiomyocyte-like cells -seeded scaffold (n = 12), the PLGA-BMMSCs group received BMMSCs-seeded scaffold (PB group) (n = 12), and the control group received a cell-free scaffold (n = 12). All procedures were performed in accordance with protocols approved by the Fourth Military Medical University Animal Research Committee and international research animal care guidelines. Peritoneal implantation of the cell-scaffold graft was performed 3 days after cell seeding in the PLGA scaffold and cultivation in vitro. The rats were anesthetized with pentobarbital (3%). Under sterile conditions, a small incision was made in the shaved abdominal wall and a pocket was formed. Then, a cell seeded or empty scaffold was placed inside the peritoneal pocket of each animal. The abdomen was closed by muscle and skin sutures and the animal was monitored until completely recovered from the anesthesia. Four weeks following implantation the grafts were harvested, fixed using 4% formaldehyde, paraffin-embed and sectioned for histological analysis.

#### Immunofluorescence staining of specific proteins of cardiomyocyte-like cells

To identify whether BMMSCs induced by 5-aza and AngII were differentiated to cardiomyocytes, immunofluorescence staining of troponin I (cTnI, Santa Cruz), sarcomeric a-actin (sarcomeric, Boster), and anti-connexin 43 (anti-Cx43, Boster), of cardiomyocytes were performed. Cells were fixed by 4% formaldehyde, blocked in 2% bovine serum albumin (BSA), stained by anti-troponin I (polyclonal IgG anti-goat 1:25); anti-connexin 43 (polyclonal IgG anti-rabbit 1:25); and anti-a-actin (polyclonal IgG anti-mouse 1:300). Negative controls were also employed in each analysis to delete the disturbance of the primary or secondary antibody. In addition, the cells on the stained scratches were counterstained with Hoechst 33258 to visualize their nuclei. The results were observed and photoed with fluorescence microscopy (Olympus BX-51, Japan)

#### Histological and Immunohistochemical Examination

Four weeks after implantation, recipient animals were sacrificed by pentobarbital overdose. The grafts were harvested, rinsed in PBS, fixed in 4% paraformaldehyde for 24 h, embedded in paraffin, cross-sectioned into 10-mm slices. Sections were stained with hematoxylin and eosin (H&E) for cells alignment. For immunohistochemical examination of expression of cardiac troponin I, the sections were incubated at 4°C overnight with the primary antibodies directed against troponin-I. The incubation with secondary antibody was at 37°C for 30 min. The reaction with the diaminobenzidine (DAB) reagent was 5-10 min. The sections were mounted for microscopic examination with neutral gum. Cells with brown granular DAB reaction product in the cytoplasm were considered positive for the protein.

#### Scanning electron microscopy

After 7 days of implantation, the grafts were harvested and fixed in 3% glutaraldehyde. Samples were then coated with a thin layer of gold for 5 min in a Sputter-coater JFC-1100. The specimen was viewed using a Japan S-3400N scanning electron microscope operating at a typical 5 kV accelerating voltage, 20°C and 10^-5 ^Torr.

#### Transmission electron microscopy

4 weeks post implantation, the grafts were harvested, initially fixed in 3% glutaraldehyde, postfixed in 1% osmium tetroxide and embedded in epoxy resin. Ultrathin sections cut horizontally were prepared and double stained with uranyl acetate and lead citrate. The cellular ultrastructure was observed with a JEM-2000EX transmission electron microscope (Japan).

#### Statistical analysis

Data are presented as mean ± S.D. Data were compared using standard or repeated measures ANOVA where appropriate. Pairwise comparisons were performed using a two-tailed Student's *t*-test. Differences were considered significant for *P*-values < 0.05.

## Results

### Cellular morphological alterations of BMMSCs by 5-aza and AngII treatment

**Figure 1:**
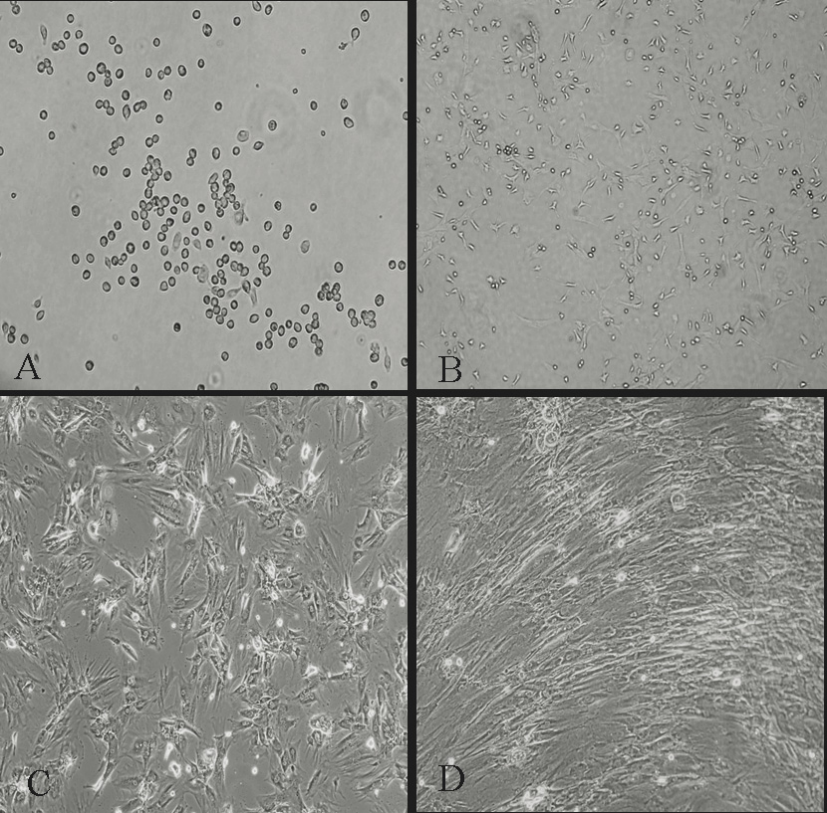
**BMMSCs' morphological alterations after inducement (A) BMMSCs cultured for 3 days (magnification: ×100)**. (B) BMMSCs cultured for 7 days (magnification: ×100). (C) BMMSCs of passage 3 (magnification: ×100). (D)Induced BMMSCs showed long spindle-shaped cells and showed uniform direction 3 weeks after induction. (Magnification: ×200).

3 days after isolation in primary culture, adhered BMMSCs presenting circular or short spindle-shaped cells with one nucleus (Figure 1A). These cells began to proliferate at about day 7, and gradually grew to form small colonies (Figure 1B). After being subcultured, the cells were polygonal or long spindle shaped (Figure 1C). The morphological differentiation from BMMSCs to cardiomyocyte-like cells evolved gradually. During exposure to 5-aza and AngII, some adherent cells died, whereas the surviving cells began to proliferate and differentiate. 1 week later, the cells showed multinucleation and extended their cytoplasmic processes with adjacent cells. Then, the cells aggregated and gradually increased in size. Three weeks later, the adherent cells were completely in contacted with adjoining cells and arranged in uniform direction (Figure 1D).

### Cardiomyocyte-specific protein expression during BMMSC differentiation

**Figure 2:**
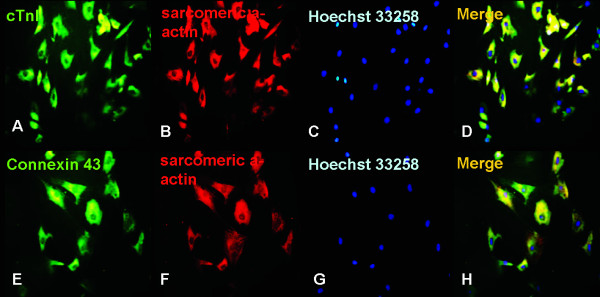
**Immunofluorescence staining for cTnI, connexin 43 and sarcomeric a-actin in induced BMMSCs**. The induced cells were positive for cardiomyocyte-specific proteins cTnI (green fluorescence, panel A, Magnification: × 200), connexin 43 (green fluorescence, panel E, Magnification: ×400) and sarcomeric a-actin (red fluorescence, panel B, F, Magnification: B: ×200, F: × 400). The nuclei were counterstained with Hoechst 33258 (blue fluorescence, panel C, G, Magnification:C: ×200, G: × 400). The panels A, B and C were merged as D(Magnification: × 200). The panels E, F and G were were merged as H(Magnification: × 400).

After induction, we evaluated the expression of cardiomyocyte-specific proteins using immunofluorescence staining. As expected, green fluorescence-labeled cTnI (Figure 2A) and connexin 43 (Figure 2E) proteins were observed in the cells by week 3 of differentiation in 5-aza and AngII. Red fluorescence-labeled sarcomeric a-actin was also observed in the induced cells (Figure 2B, F). The cells nuclei were counterstained with Hoechst 33258 (Sigma, USA) (Figure 2C, G). When cTnI proteins was merged with sarcomeric a-actin proteins, the majority of the cells showed yellow fluorescence, indicating that the cells were double-positive for both cTnI and sarcomeric a-actin proteins (Figure 2D, H).

### Macroscopic and histological characterization of the engineered myocardial tissue

**Figure 3:**
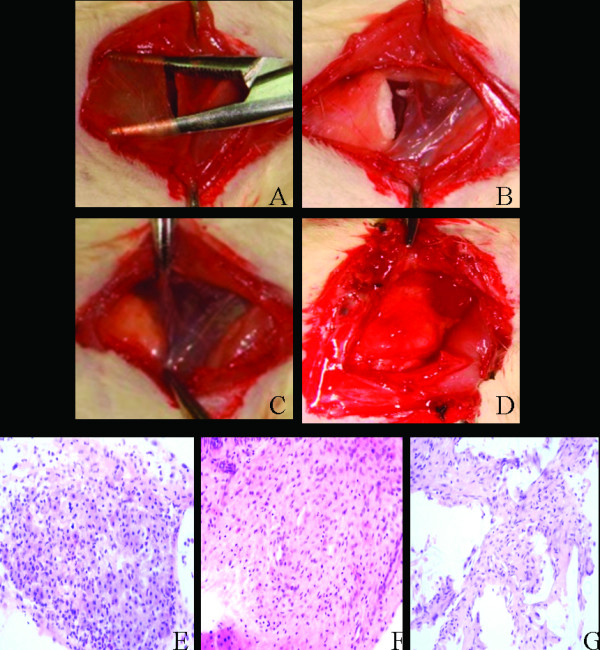
**Macroscopic and Histological analysis of the implanted tissue-constructs**. A: Preparation of the peritoneal pocket. B: EMT in situ directly after implantation. C: EMT 1 day after implantation. D: EMT 14 days after implantation. E-F: H&E staining of the tissue-constructs after implantation for 28 days in PC group, PB group and control group respectively (Magnification: × 400).

Figure 3A showed us the peritoneal pocket. Figure 3B showed us the cell seeded or empty scaffold which was placed inside the peritoneal pocket. One day after implantation, most EMTs still keep the same size and shape, only a few began to attach to greater momentum (Figure 3C). Seven days later, EMTs had almost attached to the greater momentum. Fourteen days later, the size of EMTs deflated and the shape of EMTs changed because of slight scaffolds degradation (Figure 3D). Twenty-eight days later, the EMTs had integrated with the greater omentum and about 50% scaffolds had degradated. Standard H&E staining of the implantation site 28 days after implantation showed that the cells were spread along the PLGA polymer fibers. Histological inspection of the engrafted tissue-constructs revealed the presence of differentiated forms of myocardial tissue. Moreover, some of the constructs appeared to have undergone structural maturation, although they still appeared to be less structurally mature than native cardiomyocytes. Structural maturation was manifested by the presence of an elongated pattern, apparent organized striations, and a low nucleus to cytoplasm ratio (Figure 3E). Direct differentiation from BMMSCs to the cardiomyocytes-like cells seemed few (Figure 3F). In the control group, there was no cardiomyocytes-like cells (Figure 3G). The presence of erythrocytes proved their identity as functioning blood vessels.

### Immunohistochemical analysis of the the engineered myocardial tissue

**Figure 4:**
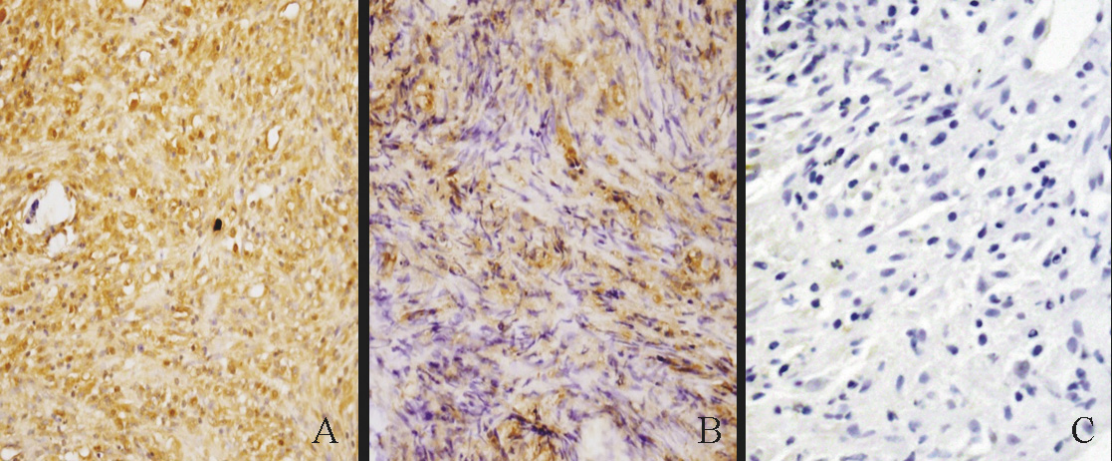
**Immunohistochemical analyses of the implanted tissue-constructs**. A-C: Immunohistochemical staining for cTnI in PC group, PBgroup and control group, respectively (Magnification: × 400).

Immunohistochemical examination showed that cells in these constructs contained many differentiated cardiac myocytes as assessed by immunohistochemical labeling of cTnI (Figure 4A), which revealed the presence of cardiomyocyte-like tissues within the implanted scaffolds in all animals. In the contrast, the cardiomyocyte-like tissues were less than those of in PB group. As can be seen from Figure 4B, the constructs only contained less than 30% differentiated cardiac myocytes. However, the constructs in control group were not positive for cTnI (Figure 4C).

### Scanning electron micrographs of the engineered myocardial tissue

**Figure 5:**
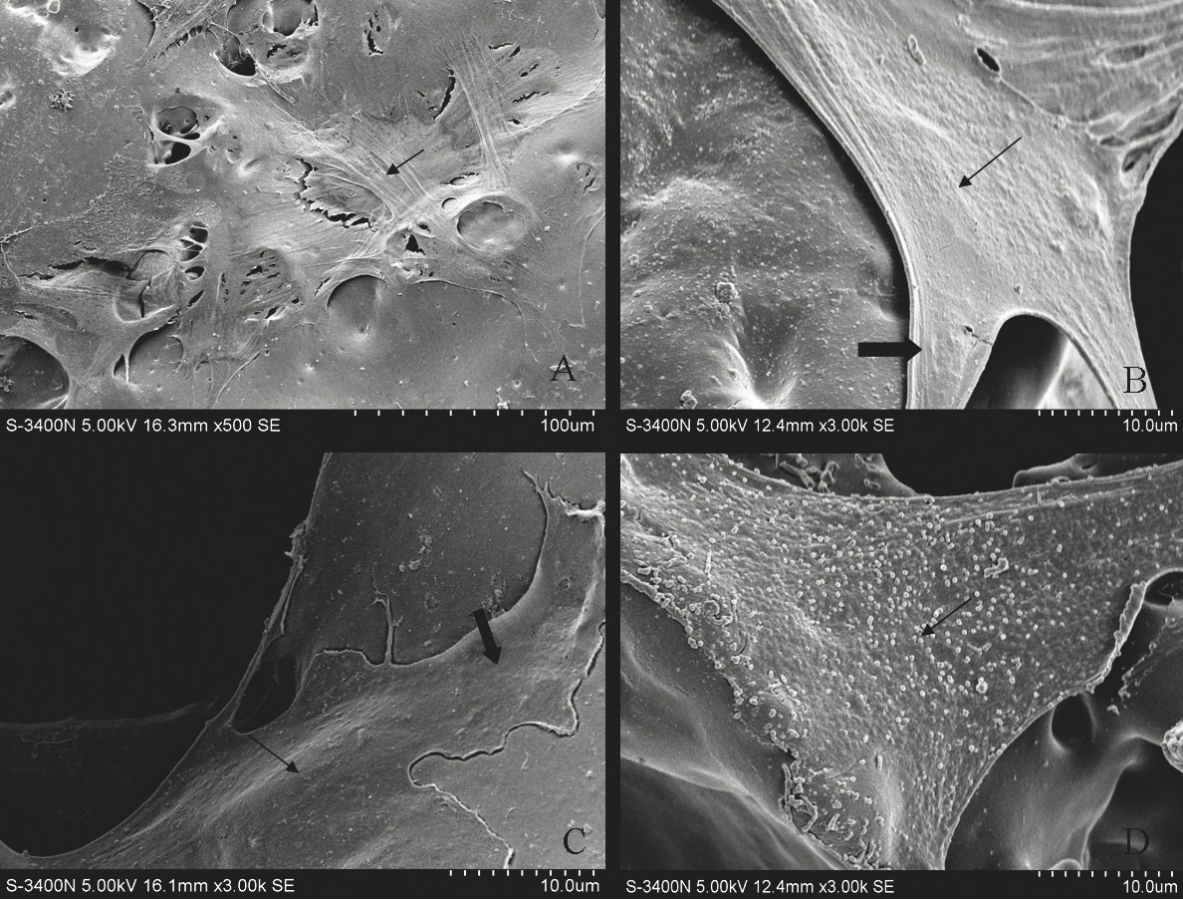
**Scanning electron microscopic images of the EMTs**. A: the cells well attached to PLGA (small arrow). B: the cell in the surface of PLGA (small arrow), and cell enations could be seen (broad arrow). C: the cell in the hole of PLGA (small arrow), and cell enations could be seen (broad arrow). D: extracellular matrix that the cells secrete (small arrow).

Scanning electron microscopy showed that the inoculated cells well attached to PLGA, and the cells grew, proliferative in 3 dimensions in constructs (Figure 5A). Many cell enations could be seen in the surface or in the hole of PLGA, which indicated that the cells had good adhesion (Figure 5B, C). In addition, the cells could secrete a lot of extracellular matrix which are essential for cell differentiation (Figure 5D).

### Ultramicrostructural characterization of the engineered myocardial tissue

**Figure 6:**
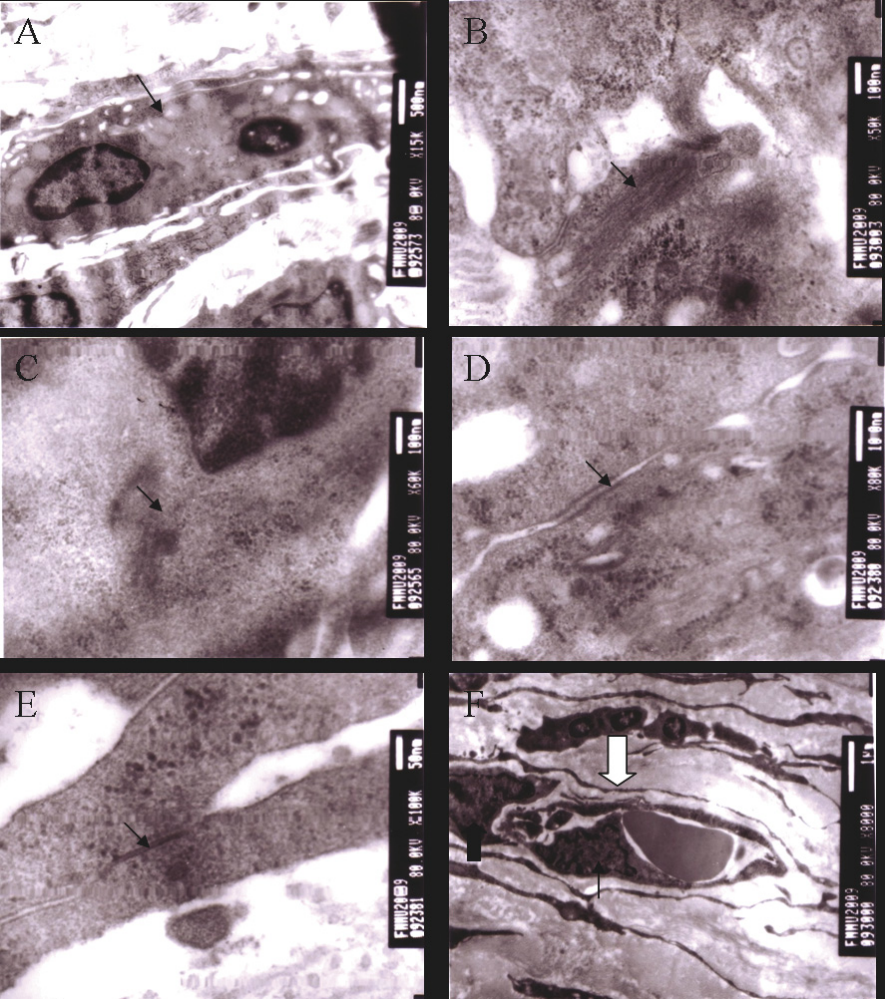
**Transmission electron microscopic images of the EMTs**. A: Representative cardiomyocyte-like cell (small arrow). B: Uniformly distributed myofilaments (small arrow). C: Z line-like substances, a distinct electron dense material (small arrow). D and E: Presence of the specialized cell- cell junctions, including the intercalated disc containing desmosomes (small arrow) and gap junctions (small arrow), characteristic of connections of heart tissue. F: Typical capillary (open arrow) composed of endothelial cells (small arrow) and a pericyte (broad arrow) is observed within the construct.

Transmission electron micrographs of constructs cultured in vivo demonstrated the presence of myocyte-like cells in PC group and PB group, but no cardiomyocyte-like cells were found in control group. The myocyte-like cells were spindle and had more than one nucleus which was located in the center of the cells (Figure 6A). In PC group, the constructs were rich in compact mitochondria and endoplasmic reticulum. Uniformly distributed myofilaments organized with clearly defined *z *line-like substances (a distinct electron dense material), which are the hallmarks of more mature cardiomyocytes, were also clearly visible (Figure 6B and 6C). In addition, we could also detect the presence of specialized junctional structures responsible for electromechanical coupling between neighboring cardiomyocytes. These included the presence of intercalated discs containing desmosomes and gap junctions (Figure 6D and 6E). And typical capillary composed of several endothelial cells and a pericyte was observed within the construct (Figure 6F). In PB group, the myocytes-like cells were fewer and were characterized by the presence of relatively disorganized myofilaments, but no desmosomes and gap junctions were found in this group. In contrast, no myofilaments, *z *line-like substances, desmosomes and gap junctions were detected in control group.

## Discussion

Myocardial tissue engineering aims at providing contractile heart muscle constructs for replacement therapy. At present, most myocardial tissue engineering attempts to utilize neonatal heart cells. However, due to limitation ability of proliferation, the heart cells can not provide the large number of the cell sources needed for engineered myocardial tissue. Over the past few years several teams have claimed that adult stem cells such as bone marrow stem cells can develop into a wide variety of cell types, including cardiomyocytes. Among them, most studies have considered bone marrow mesenchymal stem cells (BMMSCs) as a source of the "repair stem cell" [22]. BMMSCs are pluripotent cells and can produce growth factors and cytokines that play a role in their proliferation or differentiation abilities [23]. In addition, BMMSCs have immunomodulatory capabilities [24,25] as demonstrated in the field of bone marrow graft-verse-host disease [26]. Under given conditions, BMMSCs can differentiate into cells exhibiting some features of cardiomyocytes [27-30]. Makino *et al *have demonstrated that BMMSCs can differentiate into the cells with cardiac phenotype by treating with 5-azacytidine [31]. Tomita *et al *reported that the optimal concentration for cardiomyogenic differentiation is 10 μM for 24 h [32]. Others showed that ang II can induce BMMSCs into the cells with cardiac phenotype. In our study we combined these two inductors to facilitate cardiomyogenic differentiation. As a result, the period of induction decurtate to three weeks compared to that of induction only using 5-aza. Therefore, on a similar condition of induced culturing, ang II might promote the differentiation of BMMSCs induced by 5-aza.

The ideal biomaterial should be capable of being safely replaced by newly formed tissue and it should degrade at an appropriate time point without producing any toxic products [33]. PLGA scaffold has some desirable physical characteristics including high porosity, interconnected pore structure, biocompatibility and biodegradation, all of which assist in induction of tissue formation via nutritional diffusion and cell migration. In addition, PLGA can promote good cellular interaction and degrades in a set time period without toxicity. While the use of scaffolds can improve initial donor cell retention and survival following *in-vivo *engraftment by preventing cell washout and by supplying the necessary provisional matrix to which the cells can attach [34]. These scaffold characteristics should increase the survival and growth of the cells. In the present study, cardiomyocyte-like cells derived from BMMSCs were seeded on synthetic, 3-dimensional, biodegradable PLGA polymer scaffolds and cultured *in vivo*.

Vascularization is a prerequisite for successful repairing myocardial infarctions or correcting heart defects. In our study, histological analysis demonstrated the presence of red blood cells in many blood-vessels within the implanted tri-culture scaffolds, suggesting the presence of functional vasculature. These results were promising and indicated that EMTs survived after implantation *in vivo*, are vascularized and do not dedifferentiate. This is an important requisition for further experiments with implantations of EMTs on the heart and for successful clinical trial in the future. But the present experiments cannot distinct between blood vessels derived from the host and those possibly derived from EMTs. It is likely that the majority of blood vessels seen in the constructs were host vessels, but the existence of vascular structure is likely to facilitate the survival of EMTs *in vivo*. This factor could be a major advantage of the myocardial engineering tissue approach in comparison to cell transplantations.

Imaging by transmission electron microscopy revealed that EMTs are relatively uniform in terms of ultrastructure. Among the various morphological features detected, the presence of myofilaments, *z *line-like substances, and desmosomes and gap junctions might help to explain the capacity of the cells for growth and migration within EMTs in vivo [35]. Although cell/scaffold implantation can culture relatively mature EMTs, the contraction of the implanted constructs would require the electrophysiological integration with host tissue. In this respect it is interesting to note the development of gap junctions between the EMTs as well as the presence of cardiomyocyte-like cells with sarcomeric structures within the implanted scaffold.

In fact, many researchers doubt that the implanted BMMSCs are enough to differentiate into the cells with cardiac phenotype. Taking this doubt into account, we treated BMMSCs with 5-aza and angII prior to implantation studies. Our results showed that pre-treatment of BMMSCs ensure that the differentiation process will be directed towards the cardiomyogenic lineage *in vivo *environment and our findings indicate that cardiomyocyte-like cells and PLGA scaffolds in combination have synergistic effects and are more effective than BMMSCs and PLGA scaffolds or scaffolds alone.

Except for the implanted cardiomyocyte-like cells, there are some peritoneal mesothelial cells which help the growth of engineered myocardial tissues. Mesothelial cells (MCs) are accessible in human patients by excision and digestion of epiploon or from peritoneal fluid or lavage. They are easy to culture to obtain large quantities in vitro and they can be genetically modified with interesting therapeutic genes [36-38]. These cells also display angiogenic properties that could be of interest in infarct scar remodeling. The important potential of MCs in tissue engineering has recently been underlined and this cell type is probably the precursor of coronary arteries during embryogenesis [39,40]. They have already been proposed for use in patients to seed vascular prostheses [41]. MCs secrete a broad spectrum of angiogenic cytokines including SDF-1a, growth factors and extracellular matrix [42,43]. MCs are transitional mesodermal-derived cells and considered as progenitor stem cell, have similar morphological and functional properties with endothelial cells and conserve properties of transdifferentiation. MCs therapy in myocardial infarction induced neoangiogenesis in infarcted scar and preserved heart function. In conclusion, a potential therapeutic strategy would be to implant or re-implant genetically modified MCs in post-infarction injury to enhance tissue repair and healing. Imparting therapeutic target genes such as angiogenic genes would also be useful for inducing neovascularization [44]. Therefore, we think that MCs have an important role in tissue repair. In spite of these, the function of engineered myocardial tissues is also important, including the properties of heart beating. In order to test its function, the ischemic heart model is essential. We think it should not only be complicated but also need a long period to study, but we believe that the engineered myocardial tissues have potential of heart beating and repairing the damaged heart tissue. So we still need to further our study to investigate these problems in-depth in the future.

Until now, the mechanism of the formation of EMTs is still unclear. Many researchers believe that the paracrine effects of the implanted cells are more likely to influence the growth and survival of EMTs than any direct effect of the implanted cells. Nevertheless, the present results appear to indicate that tissue microenvironment in vivo is thought to play a major role in the survival and mature of EMTs, and the load in the pocket is sufficient at least to maintain the myocardial structure.

Despite these encouraging results, several obstacles need to be overcome before this strategy can become a clinical reality. These include the need to generate a directed and more efficient differentiating system to promote the differentiation rate of BMMSCs, the need to scale up the entire process to derive clinically relevant number of cardiomyocyte-like cells, and the need to establish stable culture system *in vivo *to derive more reliable and more mature engineered myocardial tissue. As other important issues, inflammation and immune response have to be considered. While inflammation caused by the surgical intervention cannot be completely avoided, an additional inflammatory response or immune response to scaffold materials or its degradation products might be existed.

## Conclusion

In summary, our study demonstrated that the cardiomyocyte -like cells derived from BMMSCs cultured on 3-dimensional PLGA scaffolds under implantation in peritoneal pocket can form engineered myocardial tissue with structural and functional features resembling those of native tissue. After 4 weeks of cultivation in vivo, engineered myocardial constructs expressed cardiac specific troponin-I. Ultrastructure of constructs indicated a differentiated cardiac myocyte phenotype including sarcomeres, desmosomes, and gap junctions. These grafts survived at least up to one month in vivo in SD rats. All of these results provide the encouraging evidence for the general feasibility of engineered myocardial tissue in vivo and support the idea that engineered myocardial tissue can be used as a model of native tissue for studies of tissue development and function and eventually for in vivo tissue repair. Despite many critical and unresolved questions, we believe that myocardial tissue engineering has a promising perspective for the replacement of malfunctioning myocardium and reconstruction of functional heart.

## Competing interests

The authors declare that they have no competing interests.

## Authors' contributions

XBY performed immunofluorescence staining. LW and WZ participated in immunohistochemical staining. YJX carried out the rest of the research, performed the statistical analysis, and drafted the manuscript. ALL and YJX conceived the study and designed the research. FC helps for the revision of the manuscript. All authors have read and approved of the final manuscript.

## References

[B1] Cohn JN Bristow MR Chien KR Colucci WS Frazier OH Leinwand LA Lorell BH Moss AJ Sonnenblick EH Walsh RA Mockrin SC Reinlib L Report of the National Heart, Lung, and Blood Institute Special Emphasis Panel on Heart Failure Research Circulation 1997 95 766 770 9054723 10.1161/01.cir.95.4.766

[B2] Murry CE Keller G Differentiation of embryonic stem cells to clinically relevant populations: Lessons from embryonic development Cell 2008 132 661 680 10.1016/j.cell.2008.02.008 18295582

[B3] Park IH Zhao R West JA Yabuuchi A Huo H Ince TA Lerou PH Lensch MW Daley GQ Reprogramming of human somatic cells to pluripotency with defined factors Nature 2008 451 141 146 10.1038/nature06534 18157115

[B4] Takahashi K Yamanaka S Induction of pluripotent stem cells from mouse embryonic and adult fibroblast cultures by defined factors Cell 2006 126 663 676 10.1016/j.cell.2006.07.024 16904174

[B5] Langer R Vacanti JP Tissue engineering Science 1993 260 920 926 10.1126/science.8493529 8493529

[B6] Christman KL Lee RJ Biomaterials for the treatment of myocardial infarction Journal of the American College of Cardiology 2006 48 907 913 10.1016/j.jacc.2006.06.005 16949479

[B7] Leor J Aboulafia-Etzion S Dar A Shapiro L Barbash IM Battler A Granot Y Cohen S Bioengineered cardiac grafts: A new approach to repair the infarcted myocardium? Circulation 2000 102 III56 61 11082363 10.1161/01.cir.102.suppl_3.iii-56

[B8] Zimmermann WH Didie M Döker S Melnychenko I Naito H Rogge C Tiburcy M Eschenhagen T Heart Muscle engineering: an update on cardiac muscle replacement therapy Cardiovascular Res 2006 71 419 429 10.1016/j.cardiores.2006.03.023 16697358

[B9] Leor J Landa N Cohen S Renovation of the injured heart with myocardial tissue engineering Expert Rev Cardiovasc Ther 2006 4 239 252 10.1586/14779072.4.2.239 16509819

[B10] Shimizu T Yamato M Isoi Y Akutsu T Setomaru T Abe K Kikuchi A Umezu M Okano T Fabrication of pulsatile Cardiac tissue grafts using a novel 3-dimensional cell sheet manipulation technique and Temperature-responsive cell culture surfaces Circ Res 2002 90 e40 10.1161/hh0302.105722 11861428

[B11] Zimmermann WH Melnychenko I Wasmeier G Didie M Naito H Nixdorff U Hess A Budinsky L Brune K Michaelis B Dhein S Schwoerer A Ehmke H Eschenhagen T Engineered heart tissue grafts improve systolic and diastolic function in infarcted rat hearts Nat Med 2006 12 452 458 10.1038/nm1394 16582915

[B12] Toma C Pittenger MF Cahill KS Byrne BJ Kessler PD Human mesenchymal stem cells differentiate to a cardiomyocyte phenotype in the adult murine heart Circulation 2002 105 93 98 10.1161/hc0102.101442 11772882

[B13] Le Blanc K Pittenger M Mesenchymal stem cells: progress toward promise Cytotherapy 2005 7 36 45 10.1080/14653240510018118 16040382

[B14] Pittenger MF Martin BJ Mesenchymal stem cells and their potential as cardiac therapeutics Circ Res 2004 95 9 20 10.1161/01.RES.0000135902.99383.6f 15242981

[B15] Muller-Ehmsen J Whittaker P Kloner RA Dow JS Sakoda T Long TI Laird PW Kedes L Survival and development of neonatal rat cardiomyocytes transplanted into adult myocardium J Mol Cell Cardio 2002 34 107 116 10.1006/jmcc.2001.1491 11851351

[B16] Balsam LB Wagers AJ Christensen JL Kofidis T Weissman IL Robbins RC Hematopoietic stem cells adopt mature hematopoietic fates in ischemic myocardium Nature 2004 428 668 673 10.1038/nature02460 15034594

[B17] Gruh I Beilner J Blomer U Schmiedl A Schmidt-Richter I Kruse ML Haverich A Martin U No evidence of transdifferentiation of human endothelial progenitor cells into cardiomyocytes after coculture with neonatal rat cardiomyocytes Circulation 2006 113 1326 1334 10.1161/CIRCULATIONAHA.105.559005 16520414

[B18] Murry CE Soonpaa MH Reinecke H Nakajima H Nakajima HO Rubart M Pasumarthi KB Virag JI Bartelmez SH Poppa V Bradford G Dowell JD Williams DA Field LJ Haematopoietic stem cells do not transdifferentiate into cardiac myocytes in myocardial infarcts Nature 2004 428 664 668 10.1038/nature02446 15034593

[B19] Li RK Jia ZQ Weisel RD Mickle DAG Choi A Yau TM Survival and function of bioengineered cardiac grafts Circulation 1999 100 II63 69 10567280 10.1161/01.cir.100.suppl_2.ii-63

[B20] Masuda S Shimizu T Yamato M Okano T Cell sheet engineering for heart tissue repair Adv Drug Deliv Rev 2008 60 277 285 10.1016/j.addr.2007.08.031 18006178

[B21] Zhang FB Li L Fang B Zhu DL Yang HT Gao PJ Passage-restricted differentiation potential of mesenchymal stem cells into cardiomyocyte-like cells Biochem Biophys Res Common 2005 336 784 792 10.1016/j.bbrc.2005.08.177 16143296

[B22] Minguell JJ Erices A Mesenchymal stem cells and the treatment of cardiac disease Exp Biol Med 2006 231 39 49 10.1177/153537020623100105 16380643

[B23] Pittenger MF Martin BJ Mesenchymal stem cells and their potential as cardiac therapeutics Circ Res 2004 95 9 20 10.1161/01.RES.0000135902.99383.6f 15242981

[B24] Rasmusson I Immune modulation by mesenchymal stem cells Exp Cell Res 2006 31 1815 1822 10.1016/j.yexcr.2006.03.019 16631737

[B25] Bolanos-Meade J Vogelsang GB Mesenchymal stem cells and organ transplantation: current status and promising future Transplantation 2006 81 1388 1389 10.1097/01.tp.0000214461.47555.70 16732174

[B26] Ringdén O Uzunel M Rasmusson I Remberger M Sundberg B Lönnies H Marschall HU Dlugosz A Szakos A Hassan Z Omazic B Aschan J Barkholt L Le Blanc K Mesenchymal stem cells for treatment of therapy-resistant graft-versus-host disease Transplantation 2006 81 1390 1397 10.1097/01.tp.0000214462.63943.14 16732175

[B27] Wang T Xu Z Jiang W Ma A Cell-to-cell contact induces mesenchymal stem cell to differentiate into cardiomyocyte and smooth muscle cell Int J Cardiol 2006 109 74 81 10.1016/j.ijcard.2005.05.072 16122823

[B28] Shim WS Jiang S Wong P Tan J Chua YL Tan YS Sin YK Lim CH Chua T Teh M Liu TC Sim E Ex vivo differentiation of human adult bone marrow stem cells into cardiomyocyte-like cell Biochem Biophys Res Commun 2004 324 481 488 10.1016/j.bbrc.2004.09.087 15474453

[B29] Siminiak T Fiszer D Jerzykowska O Grygielska B Kałmucki P Kurpisz M Percutaneous autologous myoblast transplantation in the treatment of post-infarction myocardial contractility impairment-report on two cases Kardiol Pol 2003 59 492 501 14724696

[B30] Siminiak T Grygielska B Jerzykowska O Fiszer D Kałmucki P Rzeźniczak J Kurpisz M Autologous bone marrow stem cell transplantation in acute myocardial infarction-report on two cases Kardiol Pol 2003 59 502 510 14724697

[B31] Makino S Fukuda K Miyoshi S Konishi F Kodama H Pan J Sano M Takahashi T Hori S Abe H Hata J Umezawa A Ogawa Cardiomyocytes can be generated from marrow stromal cells in vitro J Clin Invest 1999 103 697 705 10.1172/JCI5298 10074487 PMC408125

[B32] Tomita S Li RK Weisel R Mickle DA Kim EJ Sakai T Jia ZQ Autologous transplantation of bone marrow cells improves damaged heart function Circulation 1999 100 wsuppl IIx 247 256 10.1161/01.cir.100.suppl_2.ii-247 10567312

[B33] Jeong SI Kim SH Kim YH Jung Y Kwon JH Kim B-S Lee YM Manufacture of elastic biodegradable PLCL scaffolds for mechano-active vascular tissue engineering J Biomater Sci Polym Ed 2004 15 645 660 10.1163/156856204323046906 15264665

[B34] Lesman A Habib M Caspi O Gepstein A Arbel G Levenberg S Gepstein L Transplantation of a Tissue-Engineered Human Vascularized Cardiac Muscle Tissue Eng Part A 2009 10.1089/ten.TEA.2009.0130 19642856

[B35] Raimondo S Penna C Pagliaro P Geuna S Morphological characterization of GFP stably transfected adult mesenchymal bone marrow stem cells J Anat 2006 208 3 12 10.1111/j.1469-7580.2006.00511.x 16420374 PMC2100180

[B36] Murphy JE Rheinwald JG Intraperitoneal injection of genetically modified, human mesothelial cells for systemic gene therapy Hum Gene Ther 1997 8 1867 1879 10.1089/hum.1997.8.16-1867 9382953

[B37] Tiwari A Kidane A Punshon G Hamilton G Seifalian AM Extraction of cells for single-stage seeding of vascular-bypass grafts Biotechnol Appl Biochem 2003 38 35 41 10.1042/BA20030007 12641493

[B38] Nagy JA Shockley TR Masse EM Harvey VS Jackman RW Mesothelial cell-mediated gene therapy: feasibility of an ex vivo strategy Gene Ther 1995 2 393 401 7584114

[B39] Herrick SE Mutsaers SE Mesothelial progenitor cells and their potential in tissue engineering Int J Biochem Cell Biol 2004 36 621 642 10.1016/j.biocel.2003.11.002 15010328

[B40] Munoz-Chapuli R Gonzalez-Iriarte M Carmona R Atencia G Macias D Perez-Pomares JM Cellular precursors of the coronary arteries Tex Heart Inst J 2002 29 243 249 12484607 PMC140285

[B41] Hernando A Garcia-Honduvilla N Bellon JM Bujan J Navlet J Coatings for vascular prostheses: mesothelial cells express specific markers for muscle cells and have biological activity similar to that of endothelial cells Eur J Vasc Surg 1994 8 531 536 10.1016/S0950-821X(05)80586-9 7813716

[B42] Foussat A Balabanian K Amara A Bouchet-Delbos L Durand-Gasselin I Baleux F Couderc J Galanaud P Emilie D Production of stromal cell-derived factor 1 by mesothelial cells and effects of this chemokine on peritoneal B lymphocytes Eur J Immunol 2001 31 350 359 10.1002/1521-4141(200102)31:2<350::AID-IMMU350>3.0.CO;2-0 11180098

[B43] Oonakahara KI Matsuyama W Higashimoto I Kawabata M Arimura K Osame M SDF-1{alpha}/CXCL12-CXCR 4 axis is involved in the dissemination of NSCLC cells into pleural space Am J Respir Cell Mol Biol 2004 30 671 677 10.1165/rcmb.2003-0340OC 14672915

[B44] Elmadbouh I Michel JB Chachques JC Mesothelial cell transplantation in myocardial infarction Int J Artif Organs 2007 30 541 549 17628855 10.1177/039139880703000612

